# The Prevalence of Cigarette and E-cigarette Smoking Among Students in Central and Eastern Europe—Results of the YUPESS Study

**DOI:** 10.3390/ijerph16132297

**Published:** 2019-06-28

**Authors:** Grzegorz Marek Brożek, Mateusz Jankowski, Joshua Allan Lawson, Andrei Shpakou, Michał Poznański, Tadeusz Maria Zielonka, Ludmila Klimatckaia, Yelena Loginovich, Marta Rachel, Justína Gereová, Justyna Golonko, Ihar Naumau, Kamil Kornicki, Paulina Pepłowska, Valeriy Kovalevskiy, Asta Raskiliene, Krzysztof Bielewicz, Zuzana Krištúfková, Robert Mróz, Paulina Majek, Jakub Lubanski, Dorota Kaleta, Jarosław Pinkas, Jan Eugeniusz Zejda

**Affiliations:** 1 Department of Epidemiology, School of Medicine in Katowice, Medical University of Silesia in Katowice, Medykow 18 Str, 40-752 Katowice, Poland; 2 Canadian Centre for Health and Safety in Agriculture, College of Medicine, University of Saskatchewan, 104 Clinic Place, PO Box 23, Saskatoon, SK S7N 2Z4, Canada; 3 Department of Medicine, College of Medicine, University of Saskatchewan, 103 Hospital Drive, Saskatoon, SK S7N 0W8, Canada; 4 Department of Sports Medicine and Rehabilitation, Yanka Kupala State University of Grodno, 22 Ozheshko Str, 230023 Grodno, Belarus; 5 Department of General and Oncological Pulmonology, Norbert Barlicki Memorial Teaching Hospital No. 1, Medical University of Lodz, Kopcińskiego 22 Str, 90-153 Lodz, Poland; 6 Department of Family Medicine, Warsaw Medical University, Banacha Street 1a, 02-097 Warsaw, Poland; 7 Department of Social Pedagogy and Social Work, Krasnoyarsk State Pedagogical University named after V.P. Astafiev, A. Lebedeva 89 Str, Krasnoyarsk 660017, Russia; 8 Institute of Biology Systems and Genetic Research, Lithuanian University of Health Sciences, MLK, Eivenių 4Str, LT-50103 Kaunas, Lithuania; 9 Department of Human Physiology and Pathophysiology, Faculty of Medicine, University of Rzeszów, Kopisto 2a Str, 35-359 Rzeszow, Poland; 10 Department of Allergology and Cystic Fibrosis, Clinical Provincial Hospital No. 2, Lwowska 60 Str, 35-301 Rzeszów, Poland; 11 Department of Epidemiology, Faculty of Public Health, Slovak Medical University in Bratislava, Limbová 14 Str, 833 03 Bratislava, Slovak Republic; 12 Student’s Scientific Society at 2nd Department of Lung Diseases and Tuberculosis, Medical University of Bialystok, Żurawia 14 Str, 15-540 Bialystok, Poland; 13 Department of General Hygiene and Ecology, Grodno State Medical University, Gorkogo 80 Str, 230009 Grodno, Belarus; 14 Department of Childhood Psychology, Krasnoyarsk State Pedagogical University named after V.P. Astafiev, A. Lebedeva 89 Str, Krasnoyarsk 660017, Russia; 15 2nd Department of Lung Diseases and Tuberculosis, Medical University of Bialystok, Żurawia 14 Str, 15-540 Bialystok, Poland; 16 Department of Hygiene and Epidemiology, Medical University of Lodz, Zeligowskiego 7/9 Str, 90-752 Łódź, Poland; 17 School of Public Health, Centre of Postgraduate Medical Education, Kleczewska 61/63 Str, 01-826 Warsaw, Poland

**Keywords:** e-cigarettes, electronic cigarettes, smoking, tobacco, students

## Abstract

Electronic cigarettes (e-cigarettes) are an alternative to traditional tobacco cigarette smoking. The aim of this study was to assess the prevalence of cigarette smoking and e-cigarette use among university students from Central and Eastern Europe and to investigate personal characteristics associated with cigarette and e-cigarette smoking. A questionnaire-based cross-sectional survey was performed between 2017–2018 among university students in five European countries: Belarus, Lithuania, Poland, Russia, and Slovakia. The questionnaire included 46 questions related to the frequency and habits of traditional cigarettes and e-cigarettes use. Completed questionnaires were obtained from 14,352 students (8800 medical; aged 20.9 ± 2.4 years) with an overall response rate of 72.2%. Two-thirds of the respondents had smoked a traditional tobacco cigarette and 43.7% had used an e-cigarette. Overall current smoking status included 12.3% traditional cigarette smokers, 1.1% e-cigarette users, and 1.8% were dual users with the remainder being non-smokers. Smoking status differed between the research centres (*p* < 0.001). Females were less likely to try either cigarettes (OR = 0.83) or e-cigarettes (OR = 0.62) and were less likely to be current cigarette (OR = 0.64), e-cigarette (OR = 0.34), or dual users (OR = 0.33) than males. Perception of e-cigarettes significantly differed between smokers and non-smokers (*p* < 0.001). Among university students, cigarettes are more popular than e-cigarettes.

## 1. Introduction

Electronic cigarettes, also known as “e-cigarettes”, are an alternative to traditional tobacco cigarette smoking [1,2]. This electronic device heats a solution called an “e-liquid” to a temperature above 350 °C, generating an aerosol which is inhaled by the users [3]. Because of the short length of time that e-cigarettes have been available, approximately 10 years, the long-term health effects of e-smoking are not yet known [4]. There is debate around whether e-cigarette use can be used as a harm reduction tool [5,6]. In addition to this, there is evidence showing potential harmful effects of e-cigarette use [7,8,9,10]. Given these debates in the literature, it is important to investigate e-cigarette use and its potential impact on public health [5,6,7].

It is estimated that approximately 48.5 million Europeans have used an e-cigarette at least once, while 7.5 million Europeans currently use an e-cigarette [11]. Among e-cigarette users, the dominant group is the dual user group where a dual user is a person who simultaneously uses both e-cigarettes and tobacco cigarettes [12,13]. The prevalence of e-cigarette use throughout the European Union is still increasing [14]. Comparing the data from the 2012 Eurobarometer 385 survey [15] and the 2017 Eurobarometer 458 survey [16], the prevalence of e-cigarette use has increased from 7.2% to 15%. The prevalence of e-cigarette use is especially high among adolescents and young adults [14,17]. Comparing the data from the years 2013–2014 to data from 2010–2012, the percent of current users of e-cigarettes among adolescents (15–19 years) has increased from 5.5% (in 2010–2012) to 29.9% (in 2013–2014) [12,18]. Data from Hungary obtained from a group of 826 undergraduate students (21.7 ± 2.1 years) indicate that 24.9% had used an e-cigarette at some point (24.3% among medical and 25.3% among non-medical students; *p* = 0.1), and 0.6% were current e-cigarette users [19]. A higher percentage of current e-cigarette users (0.9%) was observed in a study that included 2883 students from Germany, Hungary and Norway [20]. However, in this study, e-cigarette use was not related to age, gender, academic year, religiosity, or the financial situation of the students [20]. Currently, there is a lack of data on the prevalence of e-cigarette use among the students from other European countries for this specific age group.

The aim of this study was to assess the prevalence of cigarette smoking and e-cigarette use among university students from Central and Eastern Europe and to investigate personal characteristics associated with cigarette and e-cigarette smoking. This analysis will form the basis of our research program investigating smoking habits (conventional and electronic) in a multicenter international study of university students. This study is the YoUng People E-Smoking Study —YUPESS study. It included centres from across Eastern and Central Europe with an overall aim of examining the prevalence and habits of cigarette and e-cigarette use as well as characteristics of those who use them.

## 2. Materials and Methods

### 2.1. Study Design and Population

The study was carried out between 2017 and 2018, as a part of an international multi-centre cross-sectional study—YoUng People E-Smoking Study (YUPESS). The study population was composed of university students in five European countries: Belarus (BY), Lithuania (LT), Poland (PL), Russia (RU) and Slovakia (SK). The choice of project locations (five countries in Central and Eastern Europe) was based on previous academic cooperation at a given site. Moreover, available evidence showed that the prevalence of e-cigarette use is especially high among young people from Eastern Europe in comparison to the EU average [11,15]. There is also a lack of epidemiological data on e-cigarette smoking among students from Eastern Europe, especially from Belarus and Lithuania. Most previous research is based on evidence from single country studies [13,21], ignoring the use among those from neighbouring countries.

Our study was based on convenience samples. Participants were recruited through universities. In each centre, a group of students from medicine was required. Additionally, each research centre was requested to recruit at least one group of students from a non-medical faculty (non-medical field of the study). Depending on the field of the study, participants were assigned to either the medical or non-medical group. All students attending school from selected faculties were eligible to be included in the research. All questionnaires were printed and delivered to each of the subjects personally by a member of the research team. Questionnaires were answered by students during classes or lectures. The study protocol suggested a goal of at least a 70% response rate.

Participation in the study was voluntary and anonymous. Participants would have the right to refuse to participate without giving a reason. Each participating centre fulfilled all the requirements of the national and local authorities in relation to human subjects legislation and when it was needed, provided the ethics approval of the local Ethics Committee. All procedures performed in studies involving human participants were in accordance with the ethical standards of the institutional and/or national research committee and with the 1964 Helsinki Declaration and its later amendments or comparable ethical standards. The study protocol was reviewed and approved by the Ethical Review Board at the Medical University of Silesia, Poland (decision number: KNW/0022/KB/205/16). The questionnaire was completely anonymous. The letter of invitation contained information that the fulfilment and return of the questionnaire implied an implicit consent to participate in the survey. The study protocol was limited only to questionnaires with any intervention procedures that needed separate conscious agreement.

### 2.2. Study Questionnaire

The research tool was an original questionnaire developed for the purpose of this study (Supplementary Material S1). For the preparation of the final version of the questionnaire, the results of a pilot study carried out on a group of 1318 students from the Medical University of Silesia in Katowice, Poland, in 2016 were used [13].

The questionnaire included 46 questions related to the frequency and habits of traditional cigarettes and e-cigarettes use. Questions also addressed safety issues (impact on health), smoking in public places, addictive potential, and the occurrence of respiratory symptoms, as well as personal motives related to smoking traditional and electronic cigarettes. In addition, the questionnaire contained questions about the effectiveness of using e-cigarettes as a tool to reduce or quit smoking. Presence of a chronic respiratory condition was based on a positive response to the following: “Has a doctor ever said you had any of the following illnesses: Asthma; asthmatic, spastic, or obstructive bronchitis, other chronic respiratory diseases?”. Cigarette or e-cigarette use was defined according to the answers to the questions: “Have you ever smoked/tried a traditional tobacco cigarette?” and “Have you ever smoked/tried an e-cigarette?”. Smoking status was based on the question: “Do you currently smoke cigarettes?” with four possible answers “Yes, I smoke traditional tobacco cigarettes” or “Yes, I use e-cigarettes” or “Yes, I smoke traditional cigarettes and e-cigarettes” or “No, I don’t smoke”.

Originally a questionnaire was developed in English. The questionnaire was then adapted to the language of each participating country with the use of standard procedures including back-translation. Wording was used to ensure appropriate meaning of the questions. Only printed versions of the questionnaire were included. Electronic versions of the questionnaire were not allowed.

Repeatability of the questionnaire was assessed. Among a group of 86 students, an identical questionnaire was conducted twice in an interval of 5–7 days. Questionnaires as well as the form of distribution, in both samples were identical. Kappa coefficients for the critical questions ranged from 0.94 to 1.0.

### 2.3. Statistical Analysis

The data were analysed with Statistica 12 Software (TIBCO Software Inc., Palo Alto, CA, USA) and SPSS version 25 (IBM, Armonk, NY, USA). Normality of distributions of continuous variables was assessed by the Shapiro-Wilk test. Statistical significance of differences between continuous variables was analysed by the independent samples t-test or analysis of variance (ANOVA). If the assumptions for these were not met, the Mann-Whitney U test or Kruskal Wallis test was used. The distribution of categorical variables was shown by frequencies and proportions along with 95% confidence intervals. Statistical testing to compare categorical variables was completed using the independent samples chi-square test.

Associations between personal characteristics (age, sex, country of residence, presence of a chronic condition) and education group with respect to ever trying a cigarette or e-cigarette were conducted using multiple logistic regression to adjust for confounders. The associations with current smoking status (non-smoker vs. traditional smoker vs. e-cigarette smoker vs. dual smoker) were conducted using multinomial multiple logistic regression. Similarly, the associations between personal characteristics, education, and current smoking status with perceptions of smoking were conducted using either binary or multinomial multiple logistic regression where appropriate. The strength of association was measured by the odds ratio (OR) and 95% confidence intervals (CI). Statistical inference was based on the criterion *p* < 0.05.

## 3. Results

Completed questionnaires were obtained from 14,352 students (8800 medical and 5552 non-medical) with an overall response rate of 72.2%. The distributions of age, sex, study field and response rate for each research centre are presented in Table 1. The average age of the respondents was 20.9 ± 2.4 years (range 18–34 years) with differences between research centres (*p* < 0.001). The group included more women (70.4%) than men (29.6%).

### 3.1. Ever Use of Cigarette and E-cigarettes and the Age of Smoking Initiation

The prevalence of cigarette and e-cigarette use is presented in Table 2. Two-thirds of the respondents had smoked a traditional tobacco cigarette. Use of an e-cigarette was declared by 43.7% of participants. The proportion of students who had used a cigarette or e-cigarette significantly differed between the research centres (*p* < 0.001). The highest percent of respondents who had smoked a cigarette was in Slovakia (76.5%), and the highest percentage of people who had used e-cigarettes was observed among students in Lithuania (56.6%). Young adults had reached for traditional cigarette at a younger age (16.0 ± 2.5 years old) than for an e-cigarette (18.2 ± 2.2 years old; *p* < 0.001). The average age of smoking and e-smoking initiation significantly differed (*p* = 0.001) between countries. Medical students were more likely to have used a cigarette compared to non-medical students (*p* < 0.001), but non-medical students were younger when they first tried cigarettes and e-cigarettes (Table 2). The overall frequency of ever cigarette or e-cigarette use differed (*p* < 0.001) between men and women, with a higher frequency of trying either type of cigarettes in men (Table 2).

### 3.2. Current Smoking Status

The majority of the group (84.8%) were non-smokers. Traditional tobacco cigarette smoking was declared by 12.3% of respondents while e-cigarettes were used by 1.1% of the participants. Simultaneous use of e-cigarettes and tobacco cigarettes (dual use) was declared by 1.8% of respondents. Smoking status differed between the research centres, sex, and presence of a chronic breathing condition (Table 3).

### 3.3. Perception of E-cigarettes among Young Adults

The perception of e-cigarettes effect on health among young adults is presented in Table 4. Only 6.0% of subjects believed that e-cigarette use is safe for health and that opinion was shared by 34.6% of e-smokers, 34% of dual users and only 4.7% of non-smokers (*p* = 0.00001). Two thirds of the respondents believe e-smoking results in addiction. Of all the participants, 65.2% believed that use of e-cigarettes in public places should be banned. However, only 29.5% of e-cigarette users supported e-smoking prohibition in public places, compared with 66.2% of those who did not use e-cigarettes (*p* < 0.001).

### 3.4. Adjusted Analyses

Results from the adjusted analyses (Table 5 and Table 6) confirmed the descriptive results. Lithuania and Poland were more likely to have participants try cigarette or e-smoking, while participants from Slovakia were more likely to try cigarette smoking and not e-smoking compared to Belarus. Participants from Russia were less likely to try e-smoking compared to those from Belarus. Participants from each country were more likely to be current cigarette smokers compared to those from Belarus and, with the exception of those from Slovakia, were also more likely to be current electronic cigarette smokers. Only those from Russia were more likely to be current dual users than those from Belarus.

Females were less likely to try either cigarette or electronic smoking and were less likely to be current cigarette, e-cigarette, or dual users than males (Table 5). Those in the medical field were less likely to try traditional cigarettes or to currently smoke traditional cigarettes (Table 5). Those with a chronic respiratory condition were more likely to be current cigarette or dual smokers (Table 5). Older students were more likely to have tried traditional cigarettes and be current cigarette smokers but less likely to have tried e-cigarettes (Table 5).

Results from analyses examining the perceptions of e-smoking are presented in Table 6. Cigarette, electronic, and dual smokers were all more likely to believe that e-cigarettes are safe as did those with a chronic respiratory condition while those from Lithuania and Poland, females, and older participants were less likely to think it was safe. Exclusive e-cigarette users and dual users were less likely to think you can become addicted to e-cigarettes while those from Lithuania, Poland, and Russia, females, and those with a chronic respiratory condition were more likely to think e-cigarettes are addictive. Cigarette smokers, e-cigarette smokers, and dual users all felt that e-smoking was both less and more addictive than traditional cigarette smoking. Females were less likely to believe that e-cigarettes were either more or less addictive than males. Those from Lithuania and Poland as well as those who were older were less likely to believe that e-cigarettes were less addictive than traditional cigarettes, while those in medical programs were more likely to believe that e-cigarettes were more addictive than traditional cigarettes. Finally, cigarette smokers, e-cigarette smokers, dual users, those from Lithuania, Slovakia and those from Poland were less likely to agree with a ban on public smoking of e-cigarettes while those in Russia, females, medical students, older students and those with more years of study were more likely to agree with a ban.

Because two countries did not include non-medical universities, we conducted a sensitivity analysis to compare the prevalence of cigarette smoking and e-cigarette use by including only medical universities. The results were consistent, and the interpretations did not differ (data not shown). In addition to this, we reran the multiple regression analyses after excluding Lithuania and Slovakia (two countries where there were no non-medical universities included). The results were, again, consistent and the interpretations did not differ (data not shown).

## 4. Discussion

In recent years, alternative nicotine delivery systems, such as e-cigarettes have gained popularity [1,7]. The groups particularly vulnerable to the use of e-cigarettes are adolescents and young adults, who are just shaping their pro-health attitudes [22]. These groups are also the most likely targets of promotional campaigns carried out by the tobacco and e-cigarette industry [23]. Currently, there is a lack of nationally representative epidemiological data on the prevalence of e-cigarette use across young adults in Europe. While the prevalence of e-cigarette use in the European Union is subject to periodic monitoring through the Eurobarometer survey [14,15,16], the data on e-smoking in non-EU members are very limited [21].

Among EU citizens aged ≥15 years, the proportion of those who have at least tried an e-cigarette has doubled within the last 5 years: from 7.2% in 2012 to 15% in 2017 [14,15,16]. In 2017, the highest percentage of people who used an e-cigarette (25%) was among participants aged 15–24 years [16]. In our study, 43.7% had used e-cigarettes, which is a much higher result than previously observed [14,15,16]. Such a high proportion of people who had tried an e-cigarette may stem from the fact that it was a group who grew up in the period when e-cigarettes entered the market and were intensively advertised. The proportion of students who had used a cigarette or e-cigarette significantly differed between the research centres, ranging from 33.4% in Russia and 34.4% in Slovakia to 55.6% in Lithuania. Similar variations in the prevalence of use of e-cigarettes in Central and Eastern Europe were observed in the Eurobarometer 2017 study [16]. In our study, men were more likely than women to have tried cigarettes and e-cigarettes, which is in line with previous reports from Europe and the United States [14,15,16,24].

Our study is in agreement with previously reported survey results suggesting that the majority of Europeans first reach for cigarettes and e-cigarettes between the ages of 15 and 18 years [15,25]. However, the age of nicotine initiation differed significantly between the countries. The earliest nicotine initiation was observed in non-EU countries (for cigarettes in Russia, and for e-cigarettes in Belarus), compared to EU members. This may be due to differences in tobacco control policies, different accessibility to tobacco and alternative tobacco products, or cultural differences between EU countries and other parts of Europe [26].

The results of our study showed that among the students, e-cigarettes are less popular than traditional cigarettes. The overall frequency of e-cigarette use was 2.9%, which is a higher than observed in the general population (1.7%) [16]. The commonly observed phenomenon is dual use (simultaneous use of a cigarette and e-cigarette) [12,13]. In our study, the dual smoking trend was also observed (about 60% of all e-cigarette users also smoked cigarettes). Despite the fact that students used e-cigarettes more often than the general population, the frequency of traditional cigarette smoking in the study population (12.3%) was almost twice as low as the average for the EU population (26%) [15,27]. This may be due to the population under study, which were university students and so not representative of the general population.

The data on e-cigarette use in individual countries are very limited, especially those concerning non-EU members. In 2016, the prevalence of e-smoking among students in Poland ranged from 3.5% [13] to 8.3% [28]. Non-medical students were more likely to reach for e-cigarettes (12.4%) than medical students (4.4%) [28]. In our study, no significant differences were observed in the likelihood of e-cigarette use between students of medical and non-medical faculties. However, medical students were less likely to currently smoke traditional cigarettes. Different attitudes towards smoking may be the result of knowledge about the health effects of smoking that students of medical schools acquire during their education. A study performed in 2015 [21] among high school students in three cities in Russian Federation revealed that 2.2% of students aged 15–18 were current (past 30-day use) e-cigarette users [21]. In the current study, the overall proportion of current e-cigarette users in Russia was 4% (1.4% exclusive e-cigarette users and 2.6% dual users) and it was the highest of all five countries participating in the study. The prevalence of cigarette and e-cigarette smoking obtained in this study differs from general European studies and those for individual countries [13,14,15,16,21,28]. Differences in the obtained results may be a consequence of the study population examined, different age groups, and research methodology.

In our study, females were less likely to try either cigarette or e-cigarette smoking and were less likely to be current cigarette, e-cigarette, or dual users than males. These findings are in line with previously observed gender differences in cigarette and e-cigarette smoking in Europe [15,16].

Interestingly, our study revealed that the subjects with a chronic respiratory condition were more likely to be current cigarette or dual smokers. It can be assumed that the presence of chronic respiratory symptoms may be the beginning of respiratory diseases, especially chronic obstructive pulmonary disease (COPD). Nicotine dependence levels can be so high that they are not able to quit traditional smoking so they reach for e-cigarettes [29]. On the other hand, this phenomenon may be the result of the perception that e-cigarettes may be less harmful to health and the acute effects after using the e-cigarette are small so that despite the presence of chronic respiratory conditions, they are able to smoke [30]. Such a high likelihood to dual use among subjects with chronic respiratory condition may also be due to the fact that in comparison to the healthy subjects, they are more likely consider e-cigarettes as safe for health.

E-cigarettes are advertised in the mass media as a safer alternative to traditional tobacco smoking. Numerous studies have reported that current smokers, cigarette users or dual users are more likely to perceive e-cigarettes as safe for health [7,11,13]. This observation was also confirmed in our study. The perceptions of e-cigarettes significantly differed between the countries. Different attitudes and perceptions towards e-cigarettes among students from the 5 research centres may result from cultural differences and the shape of anti-smoking policy in each country, especially various legal regulations in force in the European Union, and the tobacco control act in Belarus and Russia [31,32]. In the EU, e-cigarettes are regulated under the European Union’s Tobacco Products Directive, which has been in force since May 2016 [33]. The EU Directive sets rules on access by youth as well as packaging and labeling of e-cigarettes and its accessories [33]. Each member state can introduce additional restrictions in addition to those resulting from the Directive. This has occurred in Poland, where e-cigarettes are considered equivalent to traditional tobacco cigarettes [7,33]. Similarly, in Belarus, e-cigarettes are regulated as tobacco products [34]. A different approach is observed in the Russian Federation, where there is no special law in force regarding the e-cigarettes [35].

This study has a several limitations. First, our study targeted university students. This is a select group of individuals, and the results of our study cannot be generalized to the whole population. Second, the current smoking status was based on self-declared information provided via paper-based questionnaire. The authors are aware that there are different methods for assessing current smoking status. Some researchers consider experimentation in the past 30-days (even one or two puffs) as current use. However, recently published data showed that it can be an inappropriate definition of current tobacco or e-cigarette use [36]. Third, we used a convenience sample so caution should be taken when trying to generalize the results to other populations. Nevertheless, to our best knowledge, this study represents the latest update on the prevalence of ever and current e-cigarette use and perceived harmfulness among young adults from Central and Eastern Europe. In the face of significantly limited evidence on the use of e-cigarettes, especially in Belarus and Russia, our study adds to the epidemiological description of e-smoking and provides a good basis for future research including national representative samples. In the near future, more evidence is expected, as two large cross-sectional studies, based on the YUPESS protocol, have been implemented in Germany (Regensburg) and Canada (Saskatoon).

## 5. Conclusions

Among university students, cigarettes are more popular than e-cigarettes. The proportion of students who were regular e-cigarette users (either exclusively or as a dual smoker) ranged from 2.3% in Slovakia to 4% in Russia. Males were more likely to try either cigarette or e-cigarette smoking, and were more likely to be current cigarette, e-cigarette, or dual users than females, and this group should be the addressee of activities aimed at reducing smoking. Attitudes toward smoking differ between EU and non-EU citizens.

## Figures and Tables

**Table 1:** Description of the participants by country.

Variable	Overall	Belarus	Lithuania	Poland	Russia	Slovakia	*p*
	*n* = 14,352	*n* = 3895	*n* = 1128	*n* = 7324	*n* = 1290	*n* = 715	
Age, mean (years ± SD)	20.9 ± 2.4	19.3 ± 2.1	19.8 ± 1.3	21.9 ± 2.1	20.4 ± 2.2	22.5 ± 1.8	<0.001 *
Female (%, 95%CI)	70.4 (69.7–71.1)	71.0 (69.6–72.4)	78.5 (76.0–80.8)	67.3 (66.2–68.4)	79.2 (76.9–81.4)	69.1 (65.6–72.4)	<0.001 **
Male (%, 95%CI)	29.6 (28.9–30.4)	29.0 (27.6–30.4)	21.5 (19.2–24.0)	32.7 (31.6–33.8)	20.8 (18.7–23.1)	30.9 (27.6–34.4)	
Medical students (%, 95%CI)	61.3 (60.5–62.1)	31.4 (30.0–32.9)	100.0 (99.7–100.0)	71.0 (69.9–72.0)	41.1 (38.4–43.8)	100.0 (99.5–100.0)	<0.001 **
Non-medical students (%, 95%CI)	38.7 (37.9–39.5)	68.6 (67.1–70.0)	0.0 (0.0–0.0)	29.0 (27.9–30.1)	58.9 (56.2–61.6)	0.0 (0.0–0.0)	
Have a chronic breathing condition (%, 95%CI)	9.9 (9.4–10.4)	8.3 (7.5–9.2)	15.3 (13.4–17.6)	9.7 (9.1–10.4)	11.4 (9.8–13.3)	8.9 (7.1–11.3)	<0.001 **
Do not have a chronic breathing condition (%, 95%CI)	90.1 (89.6–90.6)	91.7 (90.8–92.5)	84.7 (82.4–86.7)	90.3 (89.6–90.9)	88.6 (86.8–90.2)	91.1 (88.7–92.9)	
Response rate (%; 95%CI)	72.2 (71.6–72.8)	71.2 (70.0–72.4)	86.8 (84.8–88.5)	70.1 (69.2–71.0)	79.1 (77.1–81.0)	71.5 (68.6–74.2)	

SD—standard deviation; * result of ANOVA; ** result of the Chi-square test.

**Table 2:** Prevalence of ever using a traditional cigarette or e-cigarette and mean age of first use.

Variable	*n*	Ever Cigarette Use% (95% CI)	Mean Age Cigarette Start, Years (SD)	Ever E-Cigarette Use% (95% CI)	Mean Age E-Cigarette Start, Years (SD)
Overall	14,352	66.1 (65.4–66.9)	16.0 ± 2.5	43.7 (42.9–44.5)	18.2 ± 2.2
Country					
Belarus	3895	55.0 (53.5–56.6)	15.6 ± 2.2	42.7 (41.2–44.3)	17.3 ± 2.0
Lithuania	1128	73.0 (70.4–75.6)	15.4 ± 2.2	56.6 (53.7–59.5)	17.7 ± 1.7
Poland	7324	71.2 (70.1–72.2)	16.5 ± 2.5	45.0 (43.9–46.1)	18.6 ± 2.2
Russia	1290	59.2 (56.5–61.9)	15.3 ± 2.6	33.4 (30.9–36.0)	18.2 ± 2.4
Slovakia	715	76.5 (73.2–79.4)	15.7 ± 2.5	34.4 (31.0–38.0)	19.8 ± 2.8
*p*		<0.001 *	0.001 **	<0.001 *	0.001 **
Sex					
Male	4252	69.5 (68.1–70.9)	15.8 ± 2.8	51.3 (49.8–52.8)	18.2 ± 2.4
Female	10,092	64.7 (63.8–65.6)	16.1 ± 2.3	40.5 (39.6–41.5)	18.1 ± 2.1
*p*		<0.001 *	<0.001 ***	<0.001 *	0.5 ***
University					
Medical	8800	68.9 (67.9–69.8)	16.1 ± 2.6	43.5 (42.5–44.6)	18.5 ± 2.3
Non-Medical	5552	61.8 (60.5–63.0)	15.8 ± 2.3	44.1 (42.7–45.4)	17.7 ± 2.0
*p*		<0.001 *	<0.001 ***	0.5*	<0.001 ***
Chronic breathing condition					
Present	1405	66.0 (63.5–68.5)	15.9 ± 2.6	43.5 (40.9–46.1)	18.1 ± 2.2
Absent	12,842	67.7 (66.9–68.5)	16.0 ± 2.5	45.7 (44.8–46.6)	18.2 ± 2.2
*p*		0.2	0.2	0.1	0.6

95% CI—95-percent confidence interval; SD—standard deviation; * result of Chi-square test; ** result of ANOVA; *** result of Mann-Whitney U-test.

**Table 3:** Classification of smoking status by country of residence, sex, and university training.

Variable	*n*	Cigarette % (95% CI)	E-Cigarette % (95% CI)	Dual User % (95% CI)	Non-Smoker % (95% CI)
Overall	14,352	12.3 (11.8–12.8)	1.1 (1.0–1.3)	1.8 (1.6–2.0)	84.8 (84.2–85.4)
Country					
Belarus	3895	10.3 (9.4–11.3)	0.7 (0.5–1.0)	2.0 (1.6–2.5)	87.0 (85.9–88.1)
Lithuania	1128	14.9 (12.9–17.1)	1.4 (0.9–2.3)	2.1 (1.4–3.2)	81.6 (79.2–83.7)
Poland	7324	12.9 (12.1–13.7)	1.3 (1.1–1.6)	1.5 (1.3–1.9)	84.3 (83.5–85.1)
Russia	1290	12.2 (10.6–14.2)	1.4 (0.9–2.2)	2.6 (1.9–3.7)	83.7 (81.6–85.6)
Slovakia	715	13.1 (10.9–15.8)	0.8 (0.4–1.8)	1.5 (0.9–2.7)	84.5 (81.6–87.0)
*p*		<0.001			
Sex					
Male	4252	15.5 (14.4–16.6)	2.0 (1.6–2.4)	3.2 (2.7–3.7)	79.4 (78.2–80.6)
Female	10,092	11.0 (10.4–11.6)	0.8 (0.6–1.0)	1.2 (1.0–1.5)	87.0 (86.4–87.7)
*p*		<0.001			
University					
Medical	8800	12.0 (11.3–12.7)	1.2 (1.0–1.4)	1.7 (1.5–2.0)	85.2 (84.4–85.9)
Non-Medical	5552	12.8 (11.9–13.7)	1.1 (0.8–1.4)	2.0 (1.6–2.4)	84.2 (83.2–85.1)
*p*		0.3			
Chronic breathing condition					
Present	1405	14.7 (12.8–16.6)	1.3 (0.7–1.9)	2.6 (1.8–3.4)	81.5 (79.5–83.5)
Absent	12,842	12.1 (11.5–12.7)	1.1 (0.9–1.3)	1.7 (1.5–1.9)	85.1 (84.5–85.7)
*p*		0.003			

95%CI—95-percent confidence interval; SD—standard deviation; *p*—result of Chi-square test.

**Table 4:** Perception of e-cigarette using among students.

Statement	Total *n* = 14,336 % (95% CI)	Cigarette Smokers *n* = 1762 % (95% CI)	E-Cigarette Users *n* = 162 % (95% CI)	Dual User *n* = 259 % (95% CI)	Non-Smokers *n* = 12,153 % (95% CI)	*p*
**E-cigarettes’ safety for health**						
Yes	6.0 (5.6–6.4)	8.0 (6.8–9.4)	34.6 (27.7–42.2)	34.0 (28.5–39.9)	4.7 (4.4–5.1)	<0.001
No	75.8 (75.1–76.5)	72.5 (70.3–74.5)	40.1 (32.9–47.8)	47.9 (41.9–54.0)	77.4 (76.7–78.1)	
No opinion	18.2 (17.5–18.8)	19.5 (17.7–21.4)	25.3 (19.2–32.5)	18.2 (13.9–23.3)	17.9 (17.2–18.6)	
**Possibility of becoming addicted to e-cigarettes**						
Yes	65.4 (64.6–66.2)	64.5 (62.3–66.7)	69.8 (62.3–76.3)	60.6 (54.6–66.4)	65.6 (64.8–66.4)	<0.001
No	14.1 (13.5–14.7)	11.8 (10.4–13.4)	22.2 (16.5–29.2)	29.0 (23.8–34.8)	14.0 (13.4–14.6)	
No opinion	20.5 (19.8–21.1)	23.7 (21.7–25.7)	8.0 (4.8–13.2)	10.4 (7.3–14.7)	20.4 (19.7–21.1)	
**Level of e-cigarette addiction**						
	*n* = 9412	*n* = 1161	*n* = 114	*n* = 161	*n* = 7976	
the same as traditional cigarette	65.4 (64.4–66.4)	59.3 (56.4–62.1)	39.5 (31.0–48.7)	39.8 (32.5–47.5)	67.2 (66.1–68.2)	<0.001
lower than traditional cigarette	22.9 (22.1–23.8)	23.9 (21.6–26.5)	47.4 (38.4–56.5)	40.4 (33.1–48.1)	22.1 (21.2–23.0)	
higher than traditional cigarette	11.7 (11.1–12.4)	16.8 (14.8–19.1)	13.2 (8.1–20.6)	19.9 (14.5–26.7)	10.8 (10.1–11.5)	
**E-cigarette usage in public places should be prohibited**						
No	34.8 (34.1–35.6)	49.6 (47.2–51.9)	70.4 (62.9–76.9)	70.5 (64.7–75.8)	31.5 (30.7–32.3)	<0.001
Yes	65.2 (64.4–65.9)	50.4 (48.1–52.8)	29.6 (23.1–37.1)	29.5 (24.2–35.3)	68.5 (67.7–69.4)	

95% CI—95-percent confidence interval; *p*—result of Chi-square test.

**Table 5:** Adjusted * associations between personal characteristics and smoking habits.

Variable	Ever Smoking			Current Smoking	
	Cigarette OR (95% CI)	E-Cigarette OR (95% CI)	Cigarette (Ref.: Non-Smoker) OR (95% CI)	E-Cigarette (Ref.: Non-Smoker) OR (95% CI)	Dual User (Ref.: Non-Smoker) OR (95% CI)
Country					
Belarus	1.00	1.00	1.00	1.00	1.00
Lithuania	2.27 (1.93–2.67)	1.97 (1.69–2.29)	1.81 (1.45–2.26)	2.37 (1.19–4.75)	1.30 (0.77–2.21)
Poland	1.60 (1.45–1.77)	1.44 (1.31–1.59)	1.32 (1.14–1.53)	2.18 (1.32–3.58)	0.93 (0.65–1.32)
Russia	1.08 (0.95–1.23)	0.79 (0.69–0.90)	1.31 (1.07–1.60)	2.58 (1.41–4.73)	1.70 (1.12–2.59)
Slovakia	2.01 (1.64–2.46)	1.02 (0.84–1.22)	1.50 (1.15–1.96)	1.74 (0.67–4.51)	1.07 (0.53–2.14)
Sex					
Male	1.00	1.00	1.00	1.00	1.00
Female	0.83 (0.77–0.90)	0.62 (0.58–0.67)	0.64 (0.58–0.71)	0.34 (0.25–0.47)	0.33 (0.25–0.42)
Education					
Non-medical	1.00	1.00	1.00	1.00	1.00
Medical	0.91 (0.84–1.00)	0.94 (0.87–1.03)	0.78 (0.69–0.88)	0.96 (0.65–1.41)	0.94 (0.69–1.23)
Chronic respiratory condition					
Absent	1.00	1.00	1.00	1.00	1.00
Present	1.04 (0.93–1.18)	1.05 (0.94–1.17)	1.24 (1.06–1.45)	1.07 (0.64–1.78)	1.50 (1.05–2.16)
Age	1.11 (1.08–1.14)	0.91 (0.89–0.94)	1.05 (1.02–1.08)	0.95 (0.85–1.07)	0.95 (0.87–1.04)
Year of studies	1.00 (0.97–1.04)	0.97 (0.94–1.01)	0.95 (0.90–0.99)	0.90 (0.77–1.06)	0.95 (0.84–1.08)

* Adjusted for each variable in the table.

**Table 6:** Adjusted * associations between smoking status and perceptions of e-cigarette use.

Variable	E-Cigarettes are Safe (Ref.: No) OR (95% CI)	No Opinion about E-Cigarette Safety (Ref.: No) OR (95% CI)	You can Become Addicted to E-Cigarettes (Ref.: No) OR (95% CI)	No Opinion about E-Cigarette Addiction (Ref.: No) OR (95% CI)	E-Cigarettes are Less Addictive than Cigarettes (Ref.: Same) OR (95% CI)	E-Cigarettes are More Addictive than Cigarettes (Ref.: Same) OR (95% CI)	E-Cigarette Use in Public Places Should be Prohibited (Ref.: Allowed) OR (95% CI)
Non-smokers	1.00	1.00	1.00	1.00	1.00	1.00	1.00
Cigarette smokers	1.83 (1.50–2.24)	1.17 (1.03–1.33)	1.14 (0.96–1.35)	1.38 (1.15–1.66)	1.22 (1.05–1.42)	1.72 (1.44–2.05)	0.47 (0.42–0.52)
Exclusive e-cigarette users	15.56 (10.51–23.03)	2.98 (1.99–4.46)	0.57 (0.36–0.89)	0.23 (0.12–0.45)	3.64 (2.41–5.50)	1.88 (1.02–3.44)	0.19 (0.14–0.27)
Dual users	10.50 (7.76–14.20)	1.53 (1.08–2.17)	0.50 (0.36–0.69)	0.25 (0.16–0.39)	2.77 (1.93–3.97)	3.03 (1.96–4.68)	0.20 (0.15–0.26)
Country							
Belarus	1.00	1.00	1.00	1.00	1.00	1.00	1.00
Lithuania	0.33 (0.23–0.47)	0.73 (0.60–0.89)	3.02 (2.39–3.81)	1.84 (1.41–2.40)	0.63 (0.50–0.78)	1.06 (0.79–1.44)	0.56 (0.48–0.65)
Poland	0.35 (0.29–0.44)	0.66 (0.58–0.74)	14.46 (12.20–17.15)	4.32 (3.59–5.20)	0.53 (0.46–0.62)	1.23 (0.99–1.52)	0.87 (0.79–0.97)
Russia	0.93 (0.73–1.19)	1.15 (0.99–1.34)	1.34 (1.13–1.59)	1.65 (1.38–1.98)	1.32 (1.08–1.62)	0.80 (0.55–1.17)	1.22 (1.06–1.41)
Slovakia	1.35 (0.97–1.87)	1.65 (1.32–2.05)	0.42 (0.34–0.52)	0.57 (0.44–0.73)	1.45 (1.10–1.91)	0.73 (0.44–1.21)	0.44 (0.37–0.53)
Sex							
Male	1.00	1.00	1.00	1.00	1.00	1.00	1.00
Female	0.58 (0.50–0.68)	0.79 (0.71–0.87)	1.59 (1.42–1.79)	1.40 (1.23–1.59)	0.76 (0.68–0.85)	0.79 (0.69–0.92)	1.17 (1.08–1.27)
Education							
Non-medical	1.00	1.00	1.00	1.00	1.00	1.00	1.00
Medical	0.98 (0.82–1.17)	0.67 (0.61–0.75)	1.54 (1.34–1.76)	0.95 (0.81–1.10)	0.98 (0.87–1.11)	1.19 (1.02–1.40)	1.16 (1.06–1.26)
Chronic respiratory condition							
Absent	1.00	1.00	1.00	1.00	1.00	1.00	1.00
Present	1.45 (1.16–1.80)	1.09 (0.94–1.26)	1.28 (1.06–1.55)	1.07 (0.87–1.32)	0.96 (0.82–1.14)	1.16 (0.95–1.42)	0.93 (0.83–1.05)
Age	0.92 (0.88–0.97)	0.97 (0.94–0.99)	0.97 (0.94–1.01)	1.00 (0.97–1.04)	0.94 (0.91–0.98)	0.98 (0.94–1.03)	1.10 (1.08–1.13)
Year of studies	0.99 (0.92–1.07)	0.93 (0.89–0.97)	1.05 (1.00–1.11)	1.00 (0.95–1.06)	0.98 (0.93–1.03)	0.97 (0.91–1.03)	1.05 (1.02–1.09)

* Adjusted for smoking status, country, sex, age, education, years in program, and chronic condition.

## Supplementary Materials

The following are available online at https://www.mdpi.com/1660-4601/16/13/2297/s1, S1: Study Questionnaire.
