# Supplementary material for: The Prevalence of Cigarette and E-cigarette Smoking Among Students in Central and Eastern Europe—Results of the YUPESS Study
Source: Int J Environ Res Public Health. 2019 Jun 28;16(13):2297. doi: 10.3390/ijerph16132297 (PMC6651674; doi:10.3390/ijerph16132297)
Supplement: Supplementary file 1 [file ijerph-16-02297-s001.pdf]

# Young Peoples E-Smoking Study (YUPESS)

**Dear Sir or Madam,**

**We invite you to take part in an international study on e-cigarette smoking (e-smoking) among university students. It is an international, multicenter project involving 15 centers from 10 countries (Belarus, Canada, Czech Republic, Georgia, Lithuania, Macedonia, Poland, Romania, Russia, Slovakia).**

**Electronic cigarette (e-cigarette) use is increasing in popularity, especially as an alternative to traditional tobacco cigarette smoking. At present, the impact of E-cigarette use on health is unclear. With your help we hope to better understand this relationship.**

**The aim of this study is to assess the impact of e-cigarettes on lung health. The first part of the project is a survey. It consists of 6 parts, containing a total of 46 questions addressed to both non-smokers and smokers of traditional and/or e-cigarettes.**

## I. General Information:

1. Gender:

   [ ] Female [ ] Male

2. Age:

   ………………

3. University: College/Department:

   ..................................................... ............................./..........................

4. Year of studies:

   ………………

## II. Questions regarding smoking:

5. Have you ever smoked / tried a traditional tobacco cigarette?

   [ ] Yes [ ] No

   If "Yes", at what age did you smoke your first cigarette? ........... years

6. Have you ever smoked / tried an e-cigarette?

   [ ] Yes [ ] No

   If "Yes", at what age did you smoke your first e-cigarette? .......... years

7. Do you currently smoke cigarettes?

[ ] Yes, I smoke traditional tobacco cigarettes
[ ] Yes, I use e-cigarettes
[ ] Yes, I smoke traditional cigarettes and e-cigarettes
[ ] No, I don’t smoke

7.1 If you have ever smoked, which product did you try first?

[ ] cigarettes [ ] e-cigarette

8. In the near future do you plan to start using e-cigarettes?

[ ] Yes [ ] No [ ] Already using

9. In the near future do you plan to start smoking traditional cigarettes?

[ ] Yes [ ] No [ ] Already smoking

10. Do you think you can become addicted to e-cigarettes?

[ ] Yes
[ ] No
[ ] No opinion

11. If "Yes", then:

[ ] E-cigarettes are as addictive as a traditional cigarette
[ ] E-cigarettes are less addictive than traditional cigarettes
[ ] E-cigarettes are more addictive than traditional cigarettes

12. Do you think that using e-cigarettes in public places should be banned?

[ ] Yes [ ] No

13. Do you think e-cigarettes are safe for your health?

[ ] Yes
[ ] No
[ ] No opinion

14. If "Yes", then:

[ ] E-cigarettes are completely safe for your health

[ ] E-cigarettes are safer than traditional cigarettes

[ ] E-cigarettes and traditional cigarettes are just as safe

**III. Questions regarding respiratory symptoms.**

15. Has a doctor ever said you had any of the following illnesses:

| | | |
|---|---|---|
| a) asthma | Yes [ ] | No [ ] |
| b) asthmatic, spastic or obstructive bronchitis | Yes [ ] | No [ ] |
| c) other chronic respiratory diseases | Yes [ ] | No [ ] |

(specify)............................................................

16. Do you use aerosolized / inhaled medications?

[ ] Yes [ ] No

17. Have you ever experienced, without contact with the smoke from a cigarette or e-cigarette:

| | | |
|---|---|---|
| a) wheezing or whistling in the chest | Yes [ ] | No [ ] |
| b) shortness of breath at rest | Yes [ ] | No [ ] |
| c) shortness of breath during exertion | Yes [ ] | No [ ] |
| d) dry chronic cough unrelated to infection | Yes [ ] | No [ ] |
| e) chronic sinusitis | Yes [ ] | No [ ] |
| f) chest tightness | Yes [ ] | No [ ] |
| g) impaired sense of smell | Yes [ ] | No [ ] |
| h) exercise intolerance | Yes [ ] | No [ ] |

**If you <u>do not</u> use traditional cigarettes or e-cigarettes here, thank you for completing the questionnaire and please move on to the LAST PAGE of this survey.**

**If you smoke cigarettes or e-cigarettes, please continue to the next section.**

**IV. Questions for people who smoke / smoked traditional cigarettes**

18. How long have you smoke / smoked traditional cigarettes?

............................ number of months (1 year = 12 months)

19. How many days per week do you smoke tobacco cigarettes?

[ ] Daily
[ ] 2-3 times per week
[ ] Weakly
[ ] Less than once a week

20. How many cigarettes do you smoke on average per day?

............................ number of cigarettes per day

20a. On average, how much do you spend on traditional cigarettes in a month?

............................ $ (Change to local currency zl)

21. Have you ever tried to quit smoking traditional cigarettes?

[ ] Yes
[ ] No

21a. If "Yes", how many times have you tried to quit: ...................

21b. If you have successfully quit smoking traditional cigarettes, for how many months have you not smoked: ............................ number of months (1 year = 12 months)

21c. Did you use any of the following pharmaceuticals when quitting smoking of traditional cigarettes?

[ ] Yes, I used nicotine patches
[ ] Yes, I used nicotine pills
[ ] Yes, I used nicotine gum
[ ] Yes, I used other forms of nicotine (specify)................................................
[ ] Yes, I used non-nicotine products
[ ] No

22. Are you going to quit smoking in the near future?

[ ] Yes
[ ] No
[ ] I have already quit

**V. Questions for people who smoke <u>traditional cigarettes, e-cigarettes or both types of cigarettes (dual smokers)</u>.**

23. Have you observed any of the following symptoms **<u>since you started smoking</u>** cigarettes or e-cigarettes:

| | **after a cigarette** | **after a e-cigarette** |
|---|---|---|
| a) wheezing or whistling in the chest | [ ] | [ ] |
| b) shortness of breath at rest | [ ] | [ ] |
| c) shortness of breath during exertion | [ ] | [ ] |
| d) dry chronic cough unrelated to infection | [ ] | [ ] |
| e) chronic sinusitis | [ ] | [ ] |
| f) chest tightness | [ ] | [ ] |
| g) impaired sense of smell | [ ] | [ ] |
| h) exercise intolerance | [ ] | [ ] |

24. Immediately after smoking a cigarette or e-cigarette, do you experience any of the following:

| | **after a cigarette** | **after a e-cigarette** |
|---|---|---|
| a) cough | [ ] | [ ] |
| b) shortness of breath | [ ] | [ ] |
| c) dizziness/vertigo | [ ] | [ ] |
| d) nausea | [ ] | [ ] |
| e) weakness | [ ] | [ ] |
| f) impaired sense of smell | [ ] | [ ] |
| g) chest tightness | [ ] | [ ] |
| h) headache | [ ] | [ ] |
| i) sore throat | [ ] | [ ] |
| j) bad taste in mouth | [ ] | [ ] |

25. **<u>Since you started smoking</u>**, have you had a cough?

[ ] Yes [ ] No

25a. **If "Yes", the cough was**: (please check all that apply)

| **after a cigarette** | **after an e-cigarette** |
|---|---|
| [ ] all the time | [ ] all the time |
| [ ] only during the day | [ ] only during the day |
| [ ] mostly in the evening/night | [ ] mostly in the evening/night |
| [ ] dry cough | [ ] dry cough |
| [ ] moist cough (with phlegm) | [ ] moist cough (with phlegm) |

26. Do you smoke more often during the following situations? (please check all that apply)

| **cigarette** | **e- cigarette** |
|---|---|
| [ ] workdays | [ ] workdays |
| [ ] weekends, days off work | [ ] weekends, days off work |
| [ ] holidays | [ ] holidays |
| [ ] Periods of increased studying (eg.:exams) | [ ] Periods of increased studying (eg.:exams) |
| [ ] other (specify)............................... | [ ] other (specify)............................... |

**If you did not use e-cigarettes this month, thank you for completing the survey and please move on to the LAST PAGE.**

**If you SMOKE E-CIGARETTES, please continue to the next section.**

**VI. Questions regarding smoking of e-cigarettes (this part of the questionnaire is to be completed only by people who stated in question 7 that they use an e-cigarette)**

27. How long have you been using e-cigarettes?

.............................. number of months (1 year = 12 months)

28. How many days per week do you use the e-cigarette?

[ ] Daily

[ ] 2-3 times per week

[ ] Weekly

[ ] Less than once a week

29. How many times per day do you use an e-cigarette?

.............................. number of sessions of e-smoking per day

30. How many times do you take a puff of an e-cigarette during a session of smoking?

[ ] 1-6

[ ] 7-15

[ ] 16-20 ...................... average number of drags per smoking session

[ ] more then 20

30a. On average, how much do you spend on smoking e-cigarettes in a month?

.............................. $ (Change to local currency zl)

31. Did health considerations make you reach for the e-cigarette?

[ ] Yes, my health did not allow me to smoke traditional cigarettes

[ ] Yes, but for considerations not related to traditional cigarettes

[ ] No

**If "Yes", please indicate the symptoms that prompted you to reach for e-cigarettes:**
(please check all that apply)

a) wheezing or whistling in the chest [ ]
b) shortness of breath at rest [ ]
c) shortness of breath during exertion [ ]
d) dry chronic cough unrelated to infection [ ]
e) chronic sinusitis [ ]
f) chest tightness [ ]
g) impaired sense of smell [ ]
h) exercise intolerance [ ]
i) other (specify)............................. ]

32. When choosing an e-cigarette (device) you **<u>mainly based this decision on</u>**:

[ ] Price
[ ] Opinion about the product (friends/Internet)
[ ] Technical specifications of the device
[ ] other (specify): …………………………………………………..

33. Why did you start using e-cigarettes? (**<u>Select max. 3</u>**)

[ ] The desire to quit smoking traditional cigarettes
[ ] Less harmful to my health
[ ] Less harmful to the health of others
[ ] Less harmful to my appearance (nails, teeth, odour)
[ ] Price
[ ] Desire to try something new
[ ] Trendy and modern appearance of e-cigarette
[ ] Unique flavours
[ ] Simplicity of operation
[ ] Family, friends have convinced me to buy
[ ] Received as a gift
[ ] Other: ……………………………………………………….

34. When selecting an e-liquid (liquid for e-cigarette) what are important criteria**:** (**Select max. 2**)

[ ] Concentration of nicotine
[ ] Price
[ ] Taste
[ ] Smell
[ ] Opinion about the product (friends/Internet)
[ ] Chemical composition

35. Where do you **most frequently** buy refills (e-liquid) for e-cigarettes?? (**Select max. 2**)

[ ] Convenience Store (eg. 7-11 – change to local store name)
[ ] Booth in a shopping mall
[ ] A store that sells e-cigarette products specifically
[ ] Internet store
[ ] Auction sites (eg. e-bay – change to local auction site)

36. Can you determine the exact amount of nicotine in the e-liquid you use **most frequently**?

[ ] I pay no attention to that
[ ] Nicotine free 0 mg
[ ] Under 8 mg
[ ] 8-16 mg ............... mg (average concentration of nicotine in e-liquid)
[ ] 16 mg and more

37. Since you started using an e-cigarette, the average content of nicotine in e-liquid:

[ ] Increased (on average by how much)................ mg
[ ] Decreased (on average by how much).................. mg
[ ] No changes, I use e-liquid with a constant level of nicotine

38. What flavour of e-liquid do you choose **most often**:

[ ] tobacco
[ ] mint/menthol
[ ] fruit
[ ] other (specify)..........................................

39. When do you usually use the e-cigarette? (**Select max. 2**)?

[ ] To relax
[ ] Spending time with friends
[ ] During a party or social event
[ ] In every free moment
[ ] In a situation where I would normally smoke traditional cigarettes
[ ] In a situation where I have no access to a traditional cigarette

40. Have you used traditional cigarettes since the time you started using e-cigarettes?

[ ] No, I only smoke e-cigarettes
[ ] Yes

40a. Since you started using an e-cigarette, the daily number of traditional cigarettes you smoke:

[ ] Decreased (on average by how many)........................................
[ ] No change
[ ] Increased (on average by how many)........................................
[ ] E- cigarette helped me quit smoking traditional cigarettes

41. In your opinion, compared to the traditional cigarette you reach for an e-cigarette:

[ ] More often [ ] Less often [ ] The same

42. Did you try to quit using traditional cigarettes before you started using e-cigarettes??

[ ] No, I never smoked traditional cigarettes
[ ] No, I have never tried to stop smoking traditional cigarettes (still smoking)
[ ] Yes, I tried to quit smoking, but without success
[ ] Yes, I have successfully quit smoking traditional cigarettes

43. Have you tried to quit smoking e-cigarettes?

[ ] Yes [ ] No

43a. If "Yes", how many time have you tried to quit smoking e-cigarettes: ....................

43b. If you've successfully quit smoking e-cigarettes, how long (in months) have you not smoked:

.............................. number of months (1 year = 12 months)

43c. When quitting e-cigarettes did you use pharmaceuticals?

[ ] Yes, I used nicotine patches
[ ] Yes, I used nicotine pills
[ ] Yes, I used nicotine gum
[ ] Yes, I used other forms of nicotine (specify)..................................................
[ ] Yes, I used non-nicotine formulations
[ ] No

44. In the near future you plan to quit smoking e-cigarettes?

[ ] Yes [ ] No

45. Do you think you are addicted to e-cigarettes?

[ ] Yes [ ] No

46. Do you promote the idea of smoking e-cigarettes as an alternative to traditional cigarettes to those people close to you?

[ ] Yes, I recommend to everyone
[ ] Yes, I encourage smokers of traditional cigarettes to change to e-cigarettes
[ ] No, I do not encourage, but I willingly share my experience in e-smoking
[ ] No, I do not recommend e-cigarettes to others

**Thank you for participating in the survey!**

**Your feedback is a valuable source of information that will allow us to better investigate the impact of e-cigarettes on health.**

**Dear Sir or Madam,**

**In our project, we also have more advanced questions. To further explore the impact of cigarette and e-cigarette use on respiratory function, we plan to perform spirometry and FeNO measurement, so we would appreciate it if you leave your contact information. <u>This is not necessary and you can return an anonymously completed questionnaire without giving any of the following information. Participation in the study will provide a unique opportunity for a free check of your respiratory status.</u> This study is also offered to non-smokers. We assure you that all information obtained will not be communicated without your consent.**

**Contact Details (voluntary) :**

**We are interested in any form of contact. If you do not want to give full details just leave your email.**

First Name: ………………………… Last Name: …………………………

Telephone: ………………………… e-mail: …………………………

**If you have any questions or concerns, please contact the person responsible for the project:**

**Mateusz Jankowski tel. 692 250 620 e- mail: matejankowski@gmail.com**

**Dr Grzegorz Brożek tel. (32) 2523734 e- mail: brozekg@sum.edu.pl**

**Change to local research centre contact details.**

**We will gladly to answer all your questions.**
